# The role of calcium ion release on biocompatibility and antimicrobial properties of hydraulic cements

**DOI:** 10.1038/s41598-019-55288-3

**Published:** 2019-12-13

**Authors:** Andreas Koutroulis, Sarah A. Kuehne, Paul R. Cooper, Josette Camilleri

**Affiliations:** 1 School of Dentistry, Institute of Clinical Sciences, College of Medical and Dental Sciences, University of Birmingham, Edgbaston, B5 7EG Birmingham, United Kingdom; 2 Department of Oral Sciences, Faculty of Dentistry, University of Otago, PO Box 56, Dunedin, 9054 New Zealand

**Keywords:** Cell culture, Microbiology techniques

## Abstract

Tricalcium silicate (TCS)-based materials produce calcium hydroxide as a byproduct of their hydration reaction. The present study investigated whether calcium ion release (CIR) affects their biological and antimicrobial properties when used as pulp protection materials. The effect of incorporation of micro-silica and calcium phosphate monobasic to radiopacified TCS-based materials was investigated. The commercial TCS-based Biodentine, Bio-C Pulpo, TotalFill Root Repair Material, TheraCal LC and a base/liner- ACTIVA BioACTIVE (Activa) were also evaluated. The hydration and CIR were monitored and correlated with biocompatibility and antimicrobial assessment of eluates. Overall, the additives altered the hydration and leaching profile of the prototype cements. The micro-silica inclusion resulted in a decreased long-term calcium hydroxide formation which was associated with neutralised cytotoxicity and antibacterial activity. Calcium phosphate did not alter the leaching profile, although a stronger antibacterial effect was induced. The commercial materials also had different CIR profiles. The water-based ones had higher CIR, and this was associated with stronger antimicrobial effect but not enhanced biological activity. Both TheraCal LC and Activa exhibited poor degree of conversion, low CIR, acceptable biocompatibility and moderate antibacterial activity. A positive correlation of CIR with antibacterial effectiveness was observed (0.3 < r < 0.49; p = 0.021, p = 0.011 for the two test bacterial cultures). No relation was shown between CIR and cytotoxicity (0.3 < r < 0.49; p = 0.150, p = 0.068 for the two cell cultures studied). The additives modified the CIR. The antimicrobial properties were dependent on the CIR; the cytotoxicity of the materials was unaffected.

## Introduction

Mineral trioxide aggregate has been developed and is thus considered to be the material of choice for root repair and surgical endodontic procedures^1,2^. Additionally, the use of MTA and hydraulic tricalcium silicate cements (TCS) for vital pulp therapy (VPT) has been found to increase the clinical success rates of direct and indirect pulp capping procedures considerably^3^. These hydraulic cements form calcium silicate hydrate (CSH) and calcium hydroxide (CH) in contact with water^4^. Release of calcium and hydroxyl ions promotes pulp tissue healing and mineralised tissue formation^5^ while the alkalinity contributes to microbial elimination^6^. To date, despite the well-established role of CH, the desirable dose-response release from pulp capping agents is unknown.

The new generation of TCS-based materials contains modifications aimed at eliminating the limitations of MTA and enhancing its physicochemical properties. Accordingly, pure TCS has replaced Portland cement as evidenced in Biodentine (Septodont, Saint Maur-des-Fosses, France) usage^7^. Inert, non-inducing discoloration materials such as zirconium oxide (ZO) have now substituted the bismuth oxide radiopacifier^8^.

In the new generation materials, there are also a number of additives such as calcium carbonate (CC)^9^, calcium phosphate (CP)^10^, silicon oxide (SO)^11^ or resin components^6^. Notably, CC is added to Biodentine serving as a nucleating agent^12^. The main rationale of CP-incorporation which is found in TotalFill (FKG, La Chaux-de-Fonds, Switzerland) was for the enhancement of biological activity, based on the formation of mineral structures resembling bone^13^. In BioAggregate (Verio Dental, Vancouver, Canada), SO is included to convert the CH to CSH in the longer term to strengthen the material, as initially exploited in the concrete industry^11,14^. A recently introduced pulp capping agent, Bio-C Pulpo (Angelus, Londrina, Brazil) is also reported to contain SO^15^. Furthermore, the inclusion of light curable monomers, as in TheraCal LC (Bisco, Schaumburg, USA)^16^, provides the ability to command cure the material and avoids problems with bonding composite resin to the underlying liner^17^.

The use of additives can alter the cement’s properties. Specifically, even though the beneficial effects of substitution of the Portland cement in MTA with pure calcium silicates and the use of inert radiopacifiers instead of bismuth oxide are indisputable, there is still a great controversy in the literature regarding the effect of further alterations. So far, addition of a second cementitious phase, namely calcium phosphate to TCS, inclusion of a resin matrix or incorporation of silicon oxide have been found to affect the hydration of materials and the calcium hydroxide released to various extents^6,11,18,19^. Overall, it is evident that efforts directed toward the enhancement of physico-mechanical properties of the cements, might have an adverse effect on their chemical profile. The aim of the present study was to characterise a series of commercial and prototype TCS-based materials and elucidate a correlation path between alterations in the CH release and biological and antimicrobial properties following inclusion of CP, SO or resins. The null hypotheses tested were that different additives will not affect calcium ion release (CIR) and consequently, changes in calcium release will not alter cements’ biological and antimicrobial properties.

## Methods

The following commercial and prototype materials were investigated:

- Bio-C Pulpo (BCP; Angelus, Londrina, Brazil)
- Biodentine (Septodont, Saint Maur-des-Fosses, France)
- TotalFill Root Repair Material (Totalfill; FKG, La Chaux-de-Fonds, Switzerland)
- Theracal (Theracal; Bisco, Schaumburg, USA)
- ACTIVA BioACTIVE Base/Liner (Activa; Pulpdent, Watertown, USA)
- TCS (Mineral Research Processing, Meyzieu, France) replaced with 30% ZO radiopacifier (Sigma Aldrich, Gillingham, UK) (TCS/ZO)
- TCS/ZO with 15% CP replacement (Sigma Aldrich, Gillingham, UK) in the cementitious phase (TCS-CP/ZO)
- TCS/ZO with 10 or 20% micro-silica (mS, Meyco 610, MBT-FEB, Manchester, UK) replacement of the cement (TCS-mS10/ZO, TCS-mS20/ZO respectively).

Replacements were performed by weight. Prototypes were mixed with distilled water. Materials were compacted inside round rubber moulds (9 mm-diameter, 1.2 mm-height). Following initial setting time of hydraulic cements which was determined according to the American Society for Testing and Materials (ASTM) standards for setting time of hydraulic cements (ASTM C266-18)^20^ or photo-polymerisation for Theracal and Activa, they were immersed in 5 ml of Hank’s balanced salt solution (HBSS; H6648, Sigma Aldrich, Gillingham, UK) and incubated at 37 °C. Experiments were performed in triplicate and with three parallels for the biocompatibility and antibacterial assays.

### Characterisation of materials

#### Degree of conversion (DC)

A Fourier-transform infrared spectrometer (Nicolet 6700, Thermo Scientific, Waltham, USA) coupled with micro-attenuated total reflectance crystal (Golden Gate-Diamond ATR, Specac, Kent, UK) was used. Real-time DC of Theracal and Activa were determined by applying a 1 mm-thick sample onto the crystal and light curing for 180 s. The distance between the curing light (Elipar™ DeepCure-S LED, 3M, Bracknell, UK) and the specimen was set at 1 mm. The wavelength of the curing light was 430–480 nm and the light intensity was 1,470 mW/cm^2^. Decrease in the intensity of the 1640 cm^−1^ peak corresponding to –CH=CH_2_ stretching vibration was monitored. The peak that corresponds to the aromatic rings at 1608 cm^−1^ was used as an internal reference as it does not change in intensity during photo-polymerisation.

#### Scanning electron microscopy (SEM), energy-dispersive spectroscopy (EDS) and X-ray diffraction (XRD) analysis

Following a 28-d incubation period, pellets were retrieved, vacuum desiccated and characterised. For SEM and EDS, materials were embedded in resin (EpoFix, Struers, Rotherham, UK), ground and polished (Phoenix Beta, Buehler, Coventry, UK) and imaged using SEM (EVO MA10, Carl Zeiss, Cambridge, UK) in backscatter mode. EDS was performed in selected areas.

For XRD analysis, pellets were crushed to a fine powder with an agate mortar and pestle. The diffractometer (Philips X’Pert 1 X-ray, Malvern Panalytical, Royston, UK) with a CoKa radiation at 40 mA and 45 kV, rotated between 15–70° with a 0.02° 2θ step and a 0.6 s step time. DIFFRAC.EVA software (Bruker, Billerica, USA) and International Centre for Diffraction Data database (Newtown Square, USA) served for phase identification.

### Leachate analysis

The immersion liquid was not changed at any point during the incubation period of pellets for all experiments conducted.

#### pH analysis

The pH of eluates was measured 24 h post-immersion and weekly for 28-d using a pH meter (Beckman Φ40, Beckman Coulter, Brea, USA).

#### Calcium release assessment

Pellets were weighed (0.0001 g precision) prior to immersion. One- and 28-d leachates were analysed using inductively coupled plasma-optical emission spectrometry (Optima 8000, Perkin Elmer, Waltham, USA).

#### Biocompatibility assays

The effect of fresh, 1- and 28-d leachates on the metabolic activity of two cell types was assessed using the 3-(4,5 dimethylthiazolyl-2-yl)-2,5-diphenyltetrazolium bromide (MTT) assay^21^. ATCC CCL-92 mouse 3T3 cells and primary human dental pulp cells (HDPCs) harvested from healthy root canals^22^ of two intact premolars of a 15-year old patient were used. Ethical approval was obtained from the Birmingham Community Healthcare NHS Foundation Trust (REC Ref.: 14/EM/1128, issued on 05 June 2018).

Cells were cultured in 75 cm^2^ flasks with 15 ml Dulbecco’s modified Eagle’s medium (DMEM) containing 4.5 g/l glucose, 1.5 g/l sodium bicarbonate, supplemented with 1% penicillin/streptomycin, 2 mM glutamine with 10% heat-inactivated foetal bovine serum (Biosera, East Sussex, UK) (DMEM-10FBS) at 37 °C with 5% CO_2_ in a humidified atmosphere. Cells were subcultured at 70–80% confluency and seeded in 96-well plates at 10,000 for 3T3 cells and 6,000 for HDPCs in 100 μl DMEM-10FBS and incubated for 24 h. Passages 2–7 were used.

#### Analysis in a transwell system

Cytocompatibility of materials during extraction procedure and immediately after material manipulation and placement was investigated with a transwell system consisting of a 96-well receiver plate and an adjustable cell-culture plate with a porous membrane (Milicell-96, Merck, Darmstadt, Germany).

Following the cell-seeding period, materials were placed in the wells of the receiver plate; 100 μl HBSS were transferred to each well and to empty wells for the blank control. Consequently, the cell-culture plate was placed upon the receiver plate. Following incubation for 24 h, the medium was aspirated and 100 μl MTT solution (0.5 mg/ml) was added to the wells. The MTT reagent was replaced with 100 μl dimethyl sulfoxide (Fisher Scientific, Loughborough, UK) after 4 h. Optical density (OD) was read in a microplate reader (ELx800, BioTek Instruments, Swindon, UK) at 570 nm. Results were expressed in respect to values of the blank control.

#### Cytotoxicity assessment of 1- and 28-d leachates

The International Standards for *in vitro* cytotoxicity (ISO 10993-5:2009) by indirect contact were followed^18^. After incubation for 24 h, 100 μl of the test eluate (undiluted) or 4 serial two-fold dilutions were added to each culture. Pure HBSS served as a blank (control) group. The MTT assay was performed the following day, as previously described.

#### Microbiological assays

*Streptococcus mutans* N3209 and *Lactobacillus casei* NCTC 16341 were cultured overnight (37 °C, 5% CO_2_) in Tryptic Soya Broth or Man, Rogosa & Sharpe broth (Sigma Aldrich, Gillingham, UK) respectively. Bacterial cultures were standardised to OD 1.0 at 600 nm (Jenway 7315, Cole-Parmer, Staffordshire, UK) and consequently diluted in fresh medium in a 1:4 ratio.

The minimum inhibitory concentration (MIC) assay was performed in 96-well plates by adding 150 μl of each bacterial inoculum separately and an equal amount of the test leachate, either undiluted or following 4 serial two-fold dilutions in HBSS. Plates were incubated overnight and OD of wells was determined at 600 nm in a microplate reader.

### Statistical analyses

The IBM SPSS Statistics software version 24 (IBM, Armonk, USA) was used. Prior to each statistical analysis, data were assessed for normality with the Shapiro–Wilk test. The majority of them followed the normal distribution and results were consequently processed using one-way ANOVA and Tukey post-hoc tests for multiple comparisons. Independent student’s t-tests were performed to compare leachates’ effects on cell cultures. Pearson correlation tests evaluated the relationship between mean values of calcium ion release and the respective biological and antimicrobial performance. The significance level was set at p = 0.050.

## Results

The initial setting time of test materials following which they were immersed in the HBBS medium is presented in Table 1.

**Table 1:** Mean initial setting time and standard deviation of commercial and prototype TCS-based materials expressed in hours (h) or minutes (min), following which, materials’ pellets were immersed in HBSS.

Materials	Setting time (h or min)
BCP	7 ± 1.1 min
Biodentine	12 ± 0.9 min
Totalfill	24.7 ± 0.6 h
TCS/ZO	36 ± 2 min
TCS-CP/ZO	48.7 ± 1.2 min
TCS-mS10/ZO	39.3 ± 3.1 min
TCS-mS20/ZO	53 ± 2 min

No statistical analysis was conducted in the data.

### Material characterisation

#### Extent of polymerisation

**Figure 1:**
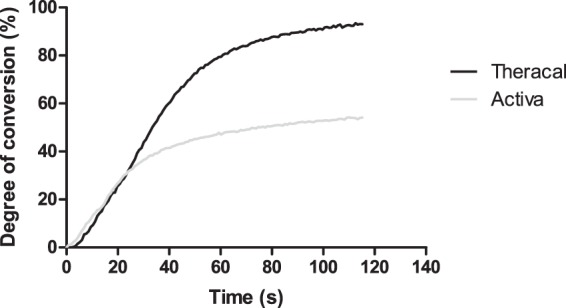
Mean degree of conversion (DC) for Theracal and Activa during exposure to light curing for a period of 120 seconds. Theracal’s DC plateaued beyond 90 s, while Activa plateaued at an earlier stage, aproximately at 60 s.

Both Activa and Theracal reached DC values below 30% during the 20-s photopolymerisation period indicated by the manufacturers. Theracal’s DC began to plateau beyond 90 s, while Activa’s DC plateaued at approximately 60 s (Fig. 1).

#### Characterisation of 28-d set materials

**Figure 2:**
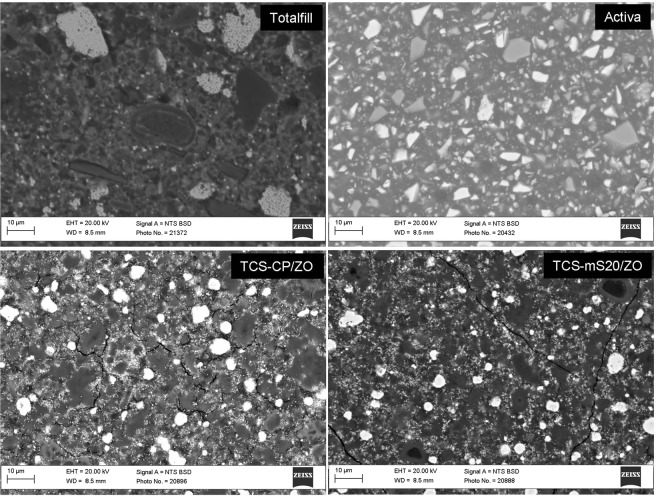
Indicative back-scatter scanning electron micrographs of 28-d aged materials (2500X magnification) showing microstructural components.

**Figure 3:**
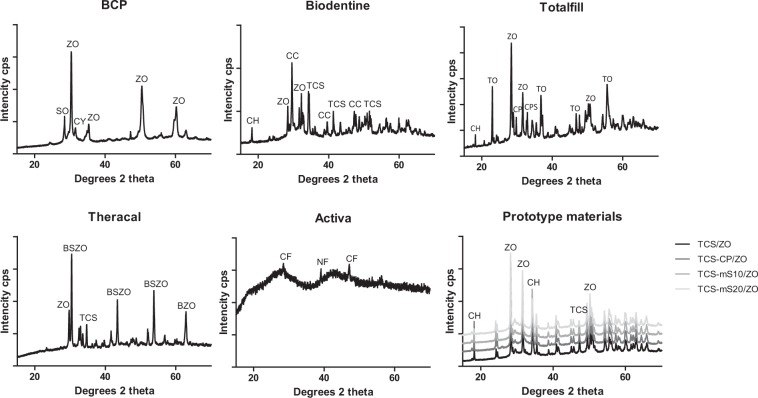
X-ray diffraction plots of test materials after a 28-d immersion period in HBSS, showing the crystalline phases formed. BSZO: Barium strontium zirconate oxide, BZO: Barium zirconate, CY: calcium hydride, CF: calcium fluoride, CPS: calcium phosphate silicate, NF: sodium fluoride, TO: tantalum oxide.

The scanning electron micrographs and energy-dispersive spectroscopy (EDS) analyses are presented in the Supplementary file. Figure 2 shows the SEM of 1 water-based commercial cement, 1 light curable one and 2 of the prototype cements (mS and CP inclusion). Figure 3 shows XRD scans. All the water-based materials tested exhibited the typical microstructure of the hydraulic-based materials with cement particles rich in calcium, silicon and oxygen interspersed in a matrix of radiopacifier and additives. The BCP, Biodentine and Totalfill contained ZO as radiopacifier with the latter also containing tantalum oxide. The EDS analysis of BCP revealed also the presence of aluminium and magnesium indicating that the cement is a Portland type. The additives included were CC (ICDD: 04-012-8783) for Biodentine, SO (ICDD: 04-002-8291) for BCP and CP (ICDD: 00-002-0647) as well as calcium phosphate silicate (ICDD: 00-050-0905) for Totalfill. The Biodentine and Totalfill exhibited the presence of CH (ICDD: 00-002-0969), while calcium hydride (ICDD: 00-001-0881) was identified in BCP.

Both light curable materials Theracal and Activa exhibited several unhydrated cement particles throughout their resin matrix (Fig. 2). Both materials had inclusions of glass phases and radiopacifiers.

The cement particles in the TCS/ZO showed a halo of hydration product. Incorporation of CP or mS altered the hydration kinetics. In the TCS-CP/ZO, the second cement type contained calcium, phosphorus and silicon and appeared lathlike. The mS-containing prototypes exhibited more advanced hydration. In the diffractograms (Fig. 3), the prototypes exhibited a similar pattern in peaks for CH (ICDD: 01-078-0315), ZO (ICDD: 00-065-0728) and TCS (ICDD: 00-003-1105). The TCS-CP/ZO and TCS-mS20/ZO compromised the intensity of the CH peaks. Lower intensity peaks for TCS were detected in the additive-containing materials.

### Leachate analysis

#### pH

**Figure 4:**
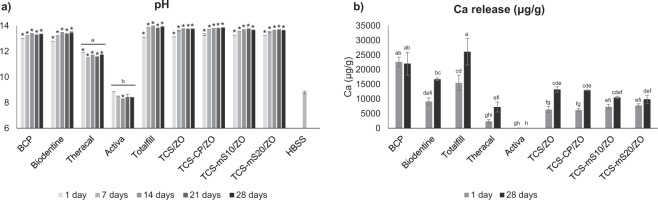
Mean pH (**a**) and calcium release (**b**) of leachates of materials for the time-points tested. Error bars indicate standard deviation. In (**a**), asterisks indicate statistical significance from the HBSS (p < 0.050) and different letters in Activa and Theracal represent signficant differences from all other eluates (p < 0.001). In (**b**), sample groups without any common letter indicate statistically significant difference (p < 0.050).

All materials alkalinised significantly the HBSS solution (p < 0.001), except for Activa, which did not present any change in the 1-d (p = 1.000), 7-d (p = 1.000), 21-d (p = 0.717) and 28-d (p = 0.811) leachates, while it exhibited a significant decrease in the pH of the 14-d leachate (p < 0.014). Alkalinisation occurred from the first day in the hydraulic cements with no significant alterations in most materials (Fig. 4a). Biodentine and TCS/ZO reported a significant increase between the 1- and 14-d leachate (p < 0.001, p = 0.030 respectively), while the pH of Totalfill increased significantly after seven days (p = 0.001). The TCS-based materials induced higher alkalinisation than Theracal (p < 0.001).

#### Calcium release

Figure 4b illustrates calcium release data. Test eluates of BCP and the 28-d leachate of Totalfill exhibited the highest values, while the former’s release plateaued after 24 h (p = 1.000). Calcium amounts in the 28-d leachate of Biodentine were not significantly different to BCP (p = 0.055). Activa released minimal amounts. Theracal’s leaching was lower than the other commercial TCS-based materials in both time periods evaluated (p < 0.001), except for Biodentine in the 1-d test leachates (p = 0.070). The CIR of mS-containing prototypes showed the same values after 24 h and at 28 d (p = 0.901 for TCS-mS10/ZO, p = 0.997 for TCS-mS20/ZO), in contrast with the behaviour of TCS/ZO and TCS-CP/ZO, where an increase was observed in the 28-d eluates (p = 0.028, p = 0.034 respectively).

#### Biocompatibility assays

**Figure 5:**
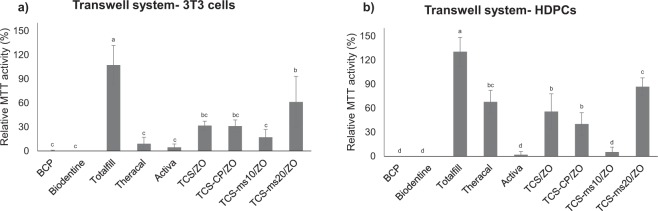
Mean relative MTT activity and standard deviation of 3T3 cell cultures (**a**) and HDPCs following a 24-h exposure to fresh leachates in a transwell system. Values appear in percentages in regards to the activity of cells in the blank control group (pure HBSS). Different letters indicate statistical difference (p < 0.050).

**Figure 6:**
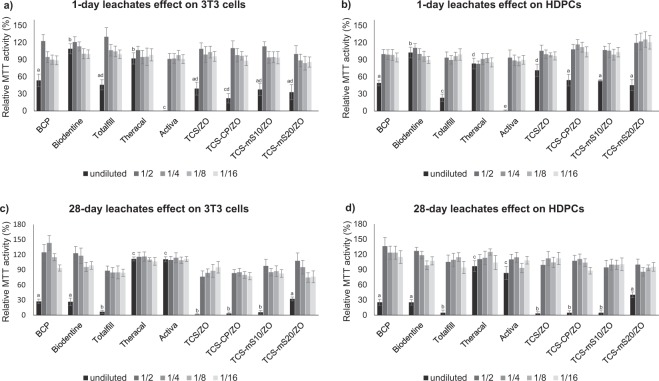
Mean relative MTT activity and standard deviation in 3T3 cell cultures and HDPC cultures after a 24-h exposure to 1-d (**a,b**) and 28-d eluates (**c,d**) of commercial and prototype materials and their dilutions. Results are expressed as percentages in regards to the MTT activity of the HBSS group. The statistical differences between non-diluted leachates are indicated with different letters in each group (p < 0.050).

Figures 5 and 6 present results of cellular metabolic activity in the transwell system and after exposure to 1- or 28-d leachates.

*Transwell system*. Totalfill was the only material that exhibited values of cell viability higher than 70% which is the threshold set by ISO 10993-5;2009^21^ in both cell types. This was distinct to all the other commercial materials (p < 0.001). Theracal exhibited increased cytotoxicity in 3T3 cultures in comparison to the HDPCs (p = 0.004). Notably, in the HDPC cultures, Theracal reported higher MTT values than BCP, Biodentine and Activa (p < 0.001), which had minimal values in both cell types (p = 1.000).

From analyses of the prototype materials, the TCS-mS20/ZO did not affect HDPC viability (mean MTT value > 70%), reporting a better performance than the unmodified prototype cement (p = 0.049). All other materials exhibited a cytotoxic potential in both cell types (MTT values < 70%). The TCS-mS10/ZO reported the lowest MTT values in the HDPCs (p < 0.001 for comparisons with TCS/ZO and TCS-mS20/ZO; p = 0.010 comparing with TCS-CP/ZO).

*One- and 28-d leachates effect*. For 1-d leachates, undiluted BCP, Totalfill, Activa and all the prototype materials reduced the metabolic activity of both cell types to different extents; undiluted Biodentine and Theracal were regarded as biocompatible (relative MTT activity > 70%), while in HDPC cultures, Biodentine reported the highest metabolic activity [p < 0.001 except for comparison with Theracal (p = 0.033)]. Totalfill exhibited increased cytotoxicity in HDPC cultures in comparison to the 3T3 ones (p < 0.001). The TCS/ZO presented a moderate decrease in MTT activity in HDPC cultures, marginally above 70%. All undiluted eluates of the modified prototype materials affected significantly more the metabolic activity in HDPC cultures in comparison to TCS/ZO (p < 0.001 for TCS-CP/ZO and TCS-mS10/ZO; p = 0.027 for TCS-mS20/ZO).

In the 28-d leachate exposed samples, all undiluted eluates were regarded as being cytotoxic (relative MTT activity < 70%) except for Theracal and Activa, which had an overall significantly better performance (p < 0.001) and reported also higher values for 3T3 cells (p = 0.010 and p < 0.001 respectively). Diluted leachates of all samples did not decrease the MTT values below the cytotoxicity margin. From the other commercial materials, Totalfill exhibited the lowest MTT values in both cell types (in 3T3 cell cultures: p = 0.005, p = 0.024; in HDPCs: p = 0.002, p = 0.007 for comparisons with BCP and Biodentine respectively). In the prototype materials, TCS-mS20/ZO exhibited better biocompatibility values in both cell types (p < 0.001). Values of the rest prototypes did not differ from the TCS/ZO (p = 1.000).

#### Antibacterial assays

**Figure 7:**
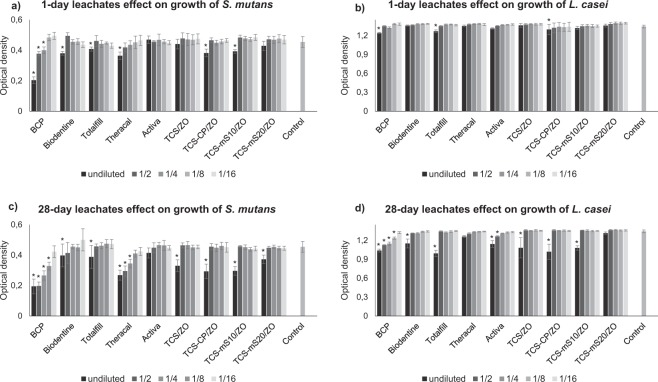
Mean optical density (OD) and standard deviation of *S. mutans* and *L. casei* cultures following overnight exposure to 1-d (**a,b**) and 28-d leachates (**c,d**) of different concentrations of materials. Asterisks indicate significant reduction of OD compared with the positive control (p < 0.010).

Results from the MIC assay against test bacteria inoculums are presented in Fig. 7. For 1-d samples of commercial materials, non-diluted leachates of BCP and Totalfill reported significantly lower optical density (OD) values than the positive control of both *S. mutans* and *L. casei* (p < 0.001 for BCP; p = 0.001 and p < 0.001 for Totalfill respectively). Notably, BCP reported higher inhibition than the other leachates (p < 0.001), except for Totalfill in the *L. casei* cultures (p = 0.997). BCP remained antibacterial even after 2 dilutions against *S. mutans* (p < 0.001). Biodentine and Theracal were antibacterial only against *S. mutans* (p < 0.001), while Activa was the only commercial material with no reported antibacterial effect (p = 1.000 for *S. mutans* and p = 0.471 in *L. casei* samples). From the prototype cements, 1-d eluates of TCS-CP/ZO reduced significantly the ODs of both *S. mutans* and *L. casei* (p < 0.001 and p = 0.002 respectively), while TCS-mS10/ZO had a demonstrable effect only against *S. mutans* (p < 0.001). TCS/ZO and TCS-mS20/ZO did not present any inhibitory effect (p = 1.000).

In the 28-d leachates, BCP exhibited the greatest inhibitory effect, reducing significantly the OD values of both bacterial inoculums in comparison with the positive control even after the third dilution (p < 0.001). All commercial materials inhibited the growth of both bacteria (p < 0.001) except for Activa, which was found antibacterial only against *L. casei* in pure eluates or after single-dilution (p < 0.001). Theracal remained antibacterial against *S. mutans* even after two dilutions (p < 0.001). From the prototype materials, TCS/ZO, TCS-CP/ZO and TCS-mS10/ZO were antibacterial against both species (p < 0.001), while TCS-mS20/ZO was effective only against *S. mutans* (p < 0.001).

### Correlation

A significant correlation between calcium ion release and antibacterial activity for both bacterial species studied was observed (0.3 < r < 0.49; p = 0.021, p = 0.011 for *S. mutans* and *L. casei* respectively). For cytotoxicity, even though a medium correlation coefficient was also reported in both cell types (0.3 < r < 0.49), overall this was non-significant (p = 0.150 for 3T3 cell cultures and p = 0.068 for HDPCs).

## Discussion

Results of the current study indicated that inclusion of additives in hydraulic cements affected calcium release to different extents, which in turn modified leachates’ biocompatibility and particularly the antibacterial reactivity. Both initial hypotheses were therefore rejected.

The majority of commercial materials tested (BCP, Biodentine and Totalfill) are composed of a TCS cement phase and radiopacifier with various additives, which have been verified in the current study. All these materials are water-based. The prototype cements were created to assess the effect of the additives on calcium ion release and the associated biological and antimicrobial properties. These prototypes did not contain any setting accelerators or other additives for enhancement of physical properties in contrast to the commercial ones. Thus, a direct correlation between inclusion of specific additives and change in properties could be explored. Theracal also contains a TCS phase but in a resin matrix. Activa was studied due to the manufacturer’s claims of bioactivity. Additionally, assessing properties of the two resin-containing materials, Theracal and Activa in the same project assisted in the evaluation of modifications of the performance of a resin-modified glass ionomer by inclusion of a cementitious phase.

Calcium release has been correlated with the biological properties of hydraulic cements, as it stimulates the differentiation potential of dental pulp cells and facilitates mineralisation leading to deposition of a dentine-like barrier upon the surface of the pulp in the long-term^23^. On the contrary, a prolonged leaching period can result in high solubility values from the material and therefore potentially compromise the restoration’s sealing ability, while minimal solubility values can affect ion release^24,25^. Despite these well-described roles, a quantitative correlation path is yet to be determined and *in vitro* values do not reflect precisely the clinical scenario^26^.

Hydroxyl ions can trigger pulp tissue healing by inducing an initial necrotic layer^5^, thus, an association of the leaching profile with the biological performance was investigated, as well as the effect of compounds on CH release. The antimicrobial activity of pulp protection materials is desirable as bacteria can remain in the dentinal tubules following pulpal infections^27^. High alkalinity provides a bactericidal effect and therefore sustained alkalinisation is desirable^28^.

Characterisation analyses performed here provided precise information on the materials’ microstructural and elemental composition as well as their mineral phases. The extent of polymerisation was assessed for the resin-containing materials as leaching of potentially cytotoxic monomers can occur if these remain unpolymerised^29^. Indirect biological and antimicrobial assays were performed to correlate the CH release with materials’ properties, without interference of materials’ surface^30^. Evaluation was performed following 1 or 28 d of immersion in HBSS to investigate the leaching profile of the hydraulic cements early during the hydration reaction and at a later stage where the final hardening and maximum physico-mechanical properties have been reached (28-d)^4^. Additionally, the immersion medium was not replaced at any point during the 28-d period since clinically the pulp capping agents are in contact with very little liquid in the application field. This resulted in leachate saturation as observed in the pH values. Biocompatibility was also assessed in a transwell system to investigate the leachates’ reactivity during the first hours of hydration.

The present study showed that calcium ion release affects positively the antibacterial effectiveness while it has a medium non-significant correlation with cytotoxicity. Therefore, an escalating reactivity was reported in leachates of TCS-based materials between the early and late stages of hydration. The relationship of the hydroxyl ions with biological properties and antimicrobial performance is also well-known^5^. However, as the pH of most TCS-based materials appeared saturated, a correlation analysis with the pH would not provide clear conclusions. Overall, calcium ions leached from hydraulic cements derive mainly from dissolution of the calcium hydroxide by-product^4^. Therefore, an increase in calcium release is also indicative of higher hydroxyl ion diffusion as well.

The BCP contains addition of SO, which is usually exploited to improve the cement’s mechanical properties^14^. Although BCP included a TCS phase, no crystalline CH was formed on hydration, however, the calcium ion release was the highest even at a relatively early stage. BCP’s 1-d leachate was reactive in terms of cytotoxicity and antibacterial performance due to its relatively high values of pH and calcium. In the transwell system, it induced cytotoxicity, providing an indication of the aggressive leaching taking place during setting reaction. The 28-d eluate exhibited increased cytotoxicity and antibacterial performance, as saturation of hydroxyl ions might have occurred.

Biodentine exhibited peaks for CH and demonstrated an overall enhanced hydration and calcium leaching profile in comparison with the chemically relevant TCS/ZO due to the presence of calcium chloride and CC in the former^19^. Biodentine’s 1-d leachate appeared reactive while the TCS/ZO exhibited moderate cytotoxicity and no bacterial inhibition. Overall, the similar performance of these materials correlates with their alkalinisation potential. Despite being both alkaline, their pH increased after 14 d. A significant cytotoxic effect could be therefore triggered after the pH exceeds a critical level^31^. Interestingly, Biodentine behaved more aggressively in the transwell system, likely due to its accelerated hydration. Besides the additives that have been tested in the current work, the commercial materials also contain calcium chloride as setting accelerator, calcium carbonate which serves as a nucleating agent or water-soluble polymers which reduce the powder to liquid ratio. This alters their chemical profile in different extents.

Totalfill exhibited a CP phase and an enhanced leaching profile which correlated with an escalating cytotoxic potential and stable antibacterial performance. Conversely, it appeared biocompatible in the transwell assay. It was observed that Totalfill remained unset during the assay period (Table 1) and therefore no significant elution from hydration by-products could have occurred in the transwell model.

The resin modification in Theracal induced significant alterations in its hydration^32^. Consequently, leaching was compromised^33^ and eluates were less reactive. The moderate alkalinisation effect following resin inclusion in TCS resulted in better biocompatibility values. However, the aggressive performance observed in the transwell assay might derive from differences in the extraction conditions which affected leaching and monomer release. Reports in the literature regarding Theracal’s biocompatibility are also controversial^6,34,35^. Interestingly, an escalating antimicrobial potential was observed against *S. mutans*, which suggests that high alkalinity is not the only factor responsible for its antimicrobial activity. Elution of unreacted monomers might contribute to the antibacterial effect^36^.

Theracal and Activa exhibited poor DC during the indicated light curing period (20 s). Under ideal light curing conditions as the ones established in the current experimental setup, the DC of a resin ranges between 50–70%^37^. For the materials tested, their shade might have compromised curing, not allowing adequate light transmission^38^. As a result, a significant amount of unreacted monomers is present in the materials following restoration.

Activa presented the typical microstructure of a glass ionomer with negligible calcium release and no alkalinisation potential. One-d and fresh eluates exhibited cytotoxicity possibly due to monomers leaching, as it has been reported previously for materials of similar composition^39,40^, albeit the manufacturer stating that Activa does not contain Bis-GMA or TEGDMA monomers^41^, which have been reported as cytotoxic^42,43^. A direct correlation between the amount of fluoride release and cytotoxicity of the glass ionomer based materials has been also suggested^44^ and should be further investigated assessing leaching of other elements in the formulation of Activa. The 28-d leachate was biocompatible, indicating that no long-term degradation of monomers occurs. In terms of antibacterial effectiveness, Activa’s inert performance was in accordance with its leaching profile.

In the prototypes, inclusion of micro-silica resulted in a plateau in calcium release at early stages and depletion of CH, especially in the TCS-mS20/ZO, which resulted in moderate reactivity. Leaching of the CP-containing material was not altered, albeit an enhanced initial antibacterial effect was observed. As high amounts of CP-inclusion compromise leaching^18^, no significant effect is reported with lower incorporation.

## Conclusions

Inclusion of additives to tricalcium silicate-based materials changed their properties. Commercial water-based hydraulic cements such as BCP, Biodentine and Totalfill exhibit increased calcium release, antibacterial activity and effect in cell metabolic activity as elution occurs throughout time. This chemical profile is compromised following a resin modification, as in Theracal, while is totally absent in a resin-based glass ionomer cement (Activa). Incorporation of silicon oxide in hydraulic cements plateaus calcium release, while inclusion of calcium phosphate in 15% does not affect significantly the calcium leaching and alkalinisation potential. Overall, calcium release is positively correlated with enhanced antimicrobial performance, while cytotoxicity is mainly affected by the alkalinity. Alterations in the traditional formulation of a radiopacified hydraulic cement in an effort to improve physical properties or handling characteristics, should be investigated as incorporation of additives may diminish the chemical, biological and antimicrobial characteristics.

## Supplementary information

Figure S1

## Data Availability

All data generated or analysed during this study are included in this published article (and its Supplementary Information files).
