# Supplementary material for: The role of calcium ion release on biocompatibility and antimicrobial properties of hydraulic cements
Source: Sci Rep. 2019 Dec 13;9:19019. doi: 10.1038/s41598-019-55288-3 (PMC6910940; doi:10.1038/s41598-019-55288-3)
Supplement: Supplementary file 1 — Figure S1 [file 41598_2019_55288_MOESM1_ESM.pdf]

The role of calcium ion release on biocompatibility and antimicrobial properties of hydraulic cements

Andreas Koutroulis, Sarah A. Kuehne, Paul R. Cooper, Josette Camilleri

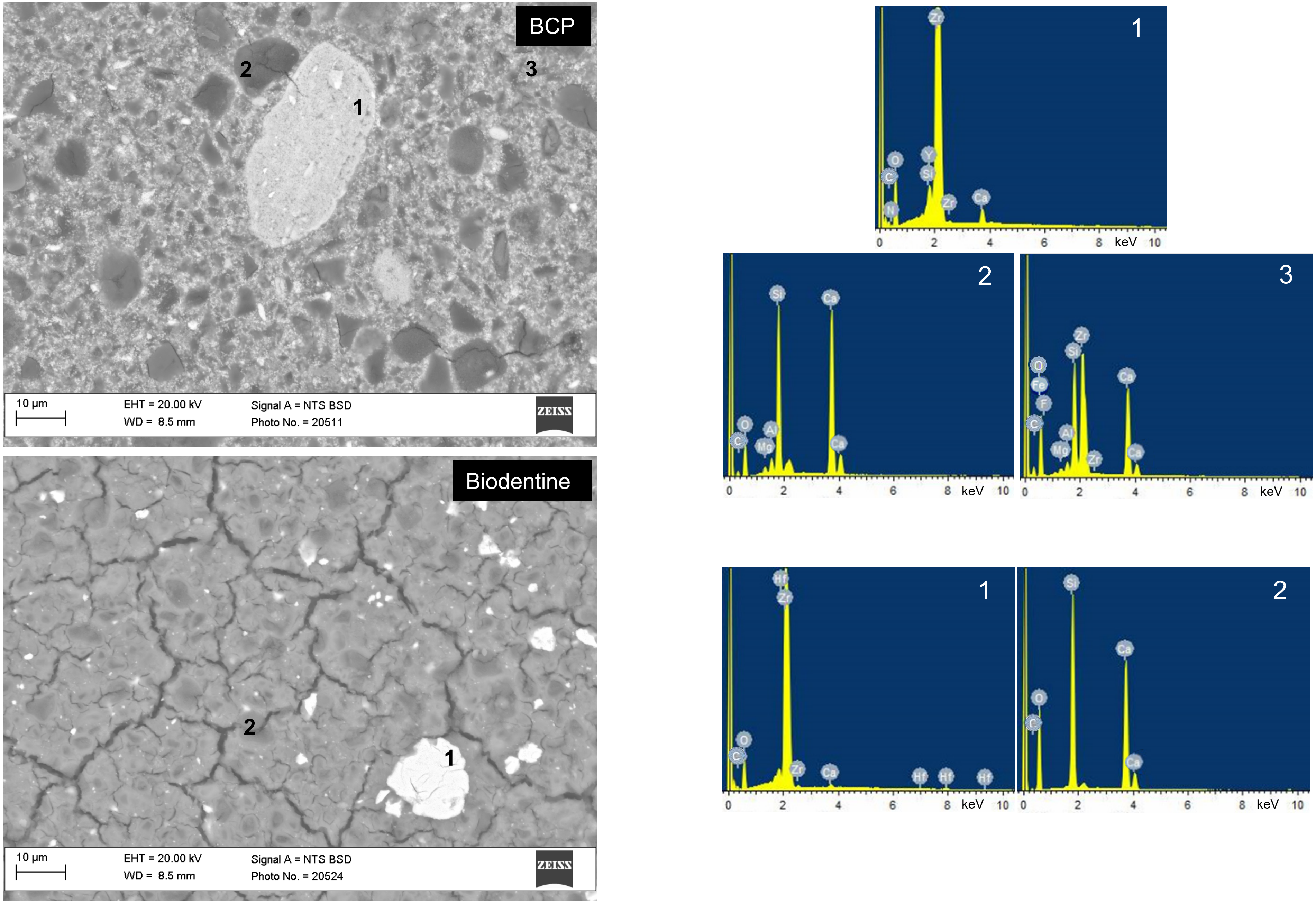
BCP
1
2
3
10 µm
EHT = 20.00 kV
WD = 8.5 mm
Signal A = NTS BSD
Photo No. = 20511
Biodentine
1
2
10 µm
EHT = 20.00 kV
WD = 8.5 mm
Signal A = NTS BSD
Photo No. = 20524
1
Zr
O
Y
C
Si
Zr
Ca
N
0 2 4 6 8 keV 10
2
Si
Ca
O
Al
C
Mg
Ca
0 2 4 6 8 keV 10
3
Zr
Si
O
Fe
F
Ca
C
Al
Mg
Ca
Zr
0 2 4 6 8 keV 10
1
Hf
Zr
O
C
Zr
Ca
Hf
Hf
Hf
0 2 4 6 8 keV 10
2
Si
Ca
O
C
Ca
0 2 4 6 8 keV 10

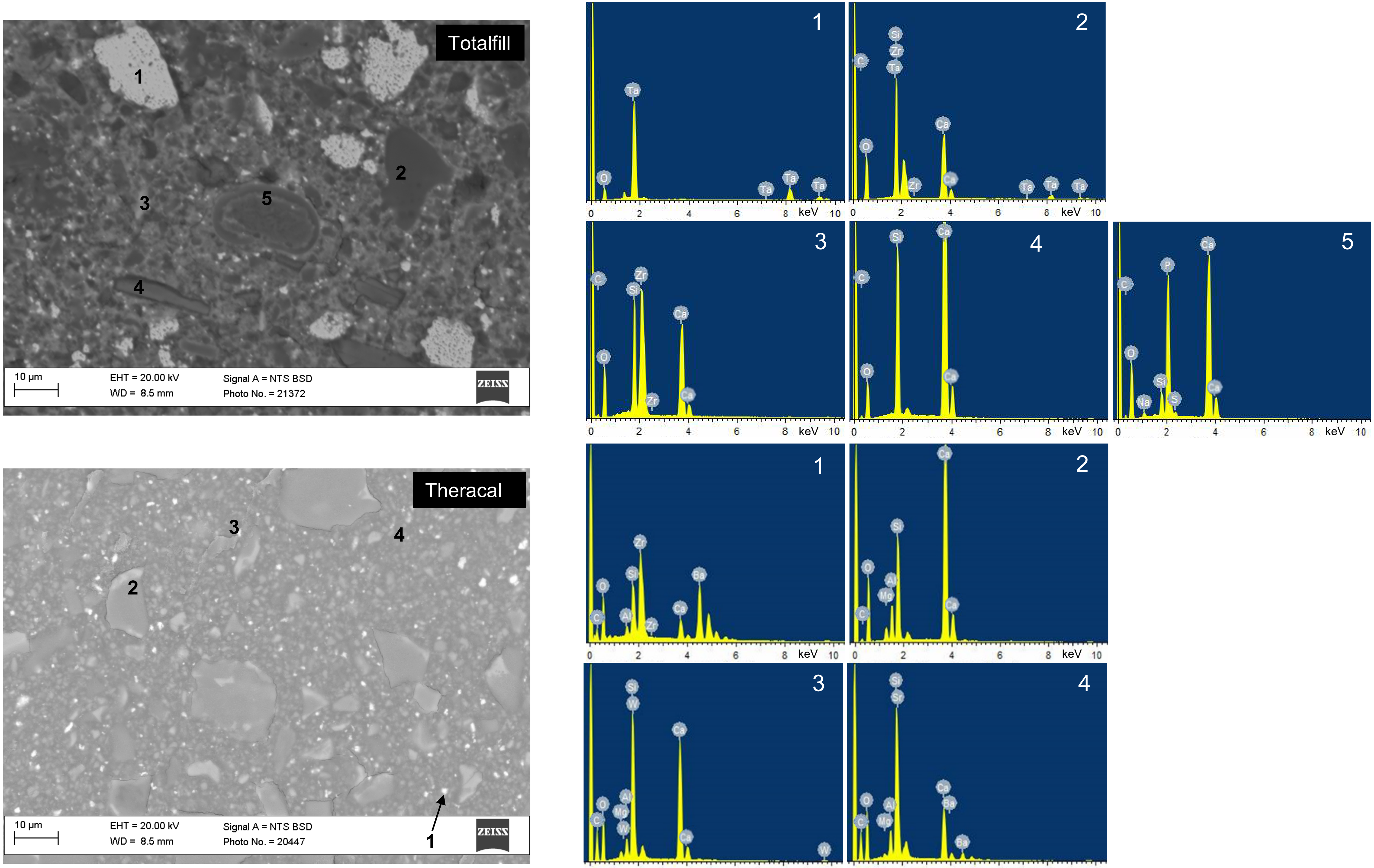
Totalfill
1
2
3
4
5
10 µm
EHT = 20.00 kV
WD = 8.5 mm
Signal A = NTS BSD
Photo No. = 21372
Theracal
1
2
3
4
10 µm
EHT = 20.00 kV
WD = 8.5 mm
Signal A = NTS BSD
Photo No. = 20447
O Ta Ta Ta
C O Si Zr Ta Zr Ca Ca Ta Ta Ta
C O Si Zr Zr Ca Ca
C O Si Ca Ca
C O Na Si P S Ca Ca
C O Al Si Zr Zr Ca Ba
C O Mg Al Si Ca Ca
C O Mg W Al Si W Ca Ca W
C O Mg Al Si Sr Ca Ba Ba
keV

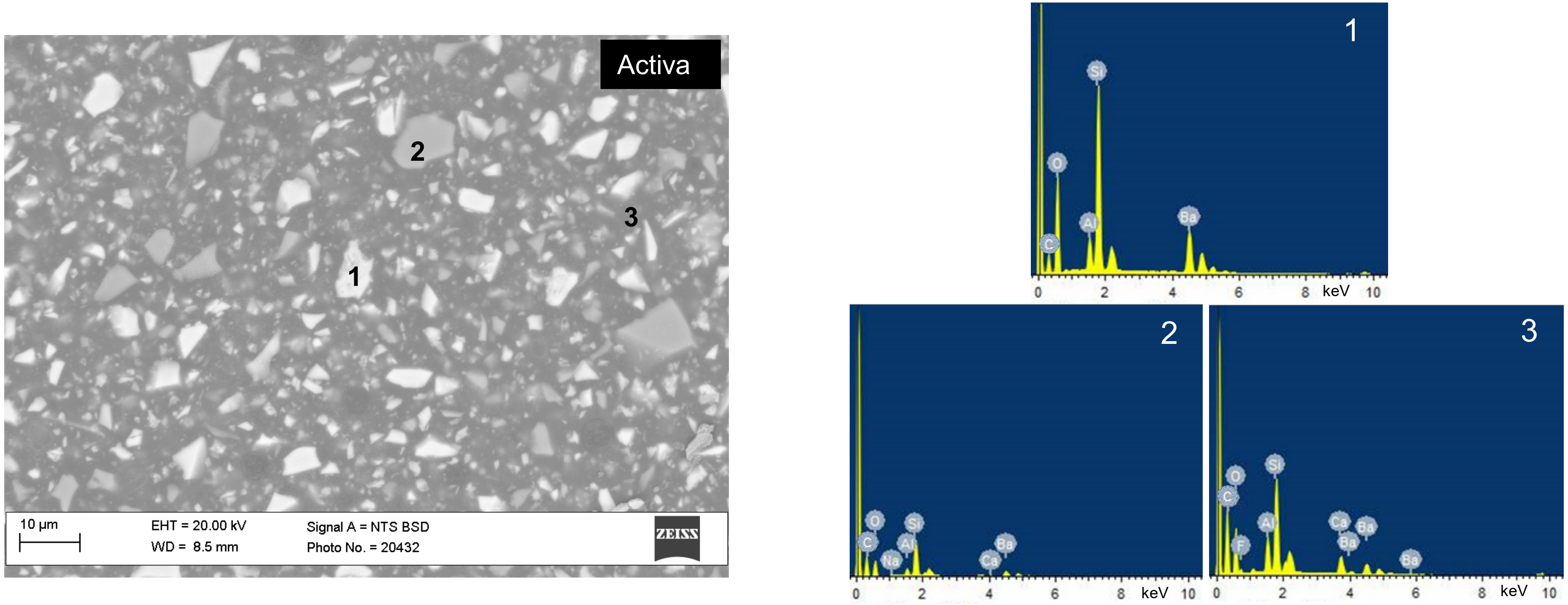
Activa
1
2
3
10 µm
EHT = 20.00 kV
WD = 8.5 mm
Signal A = NTS BSD
Photo No. = 20432
ZEISS
1
C O Al Si Ba
0 2 4 6 8 keV 10
2
C O Na Al Si Ca Ba
0 2 4 6 8 keV 10
3
C O F Al Si Ca Ba Ba Ba
0 2 4 6 8 keV 10

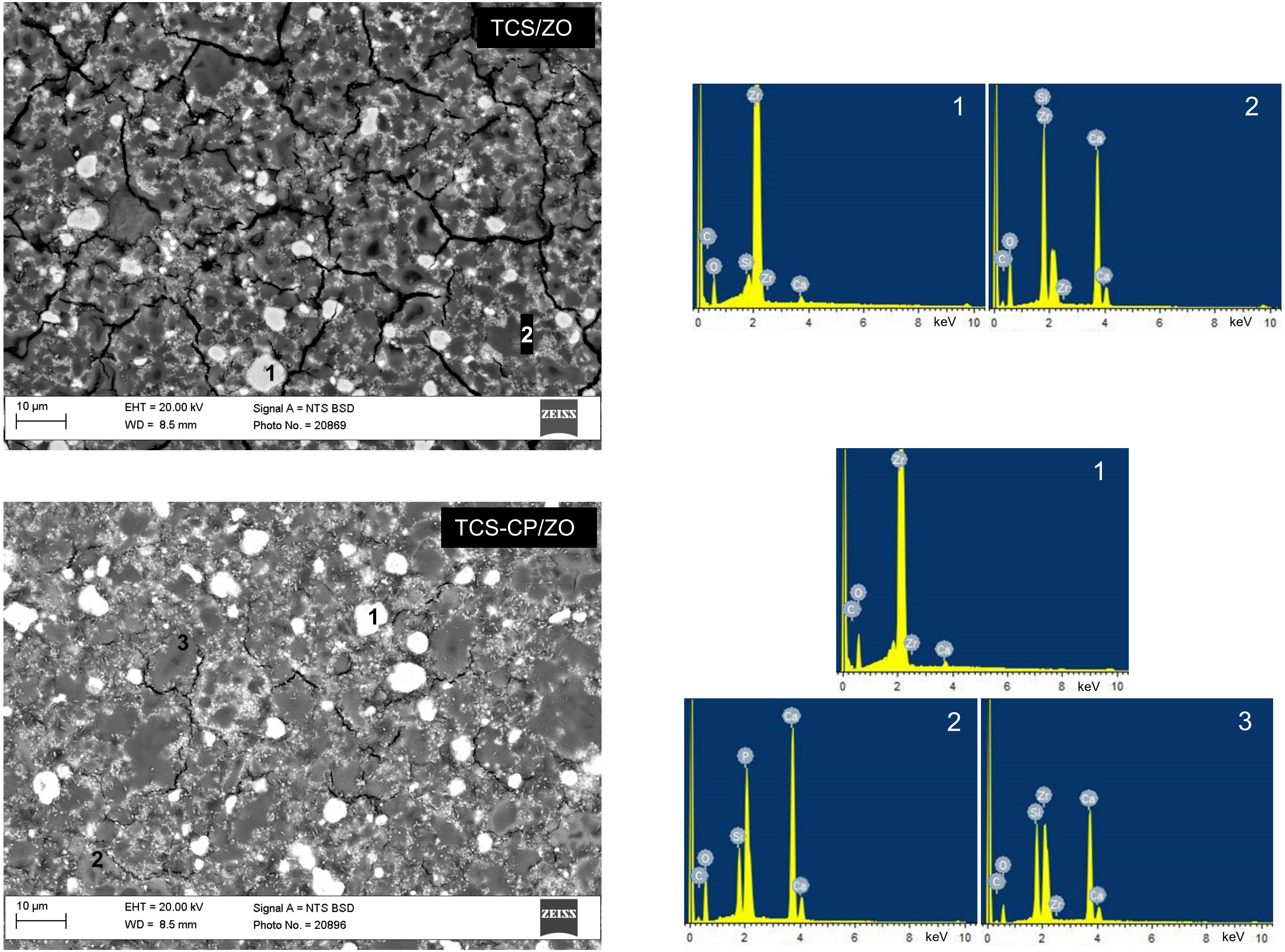
TCS/ZO
1
2
10 µm
EHT = 20.00 kV
WD = 8.5 mm
Signal A = NTS BSD
Photo No. = 20869
ZEISS
1
Zr
C
O
Si
Zr
Ca
0 2 4 6 8 keV 10
2
Si
Zr
Ca
O
C
Zr
Ca
0 2 4 6 8 keV 10
TCS-CP/ZO
1
3
2
10 µm
EHT = 20.00 kV
WD = 8.5 mm
Signal A = NTS BSD
Photo No. = 20896
ZEISS
1
Zr
O
C
Zr
Ca
0 2 4 6 8 keV 10
2
Ca
P
Si
O
C
Ca
0 2 4 6 8 keV 10
3
Zr
Si
Ca
O
C
Zr
Ca
0 2 4 6 8 keV 10

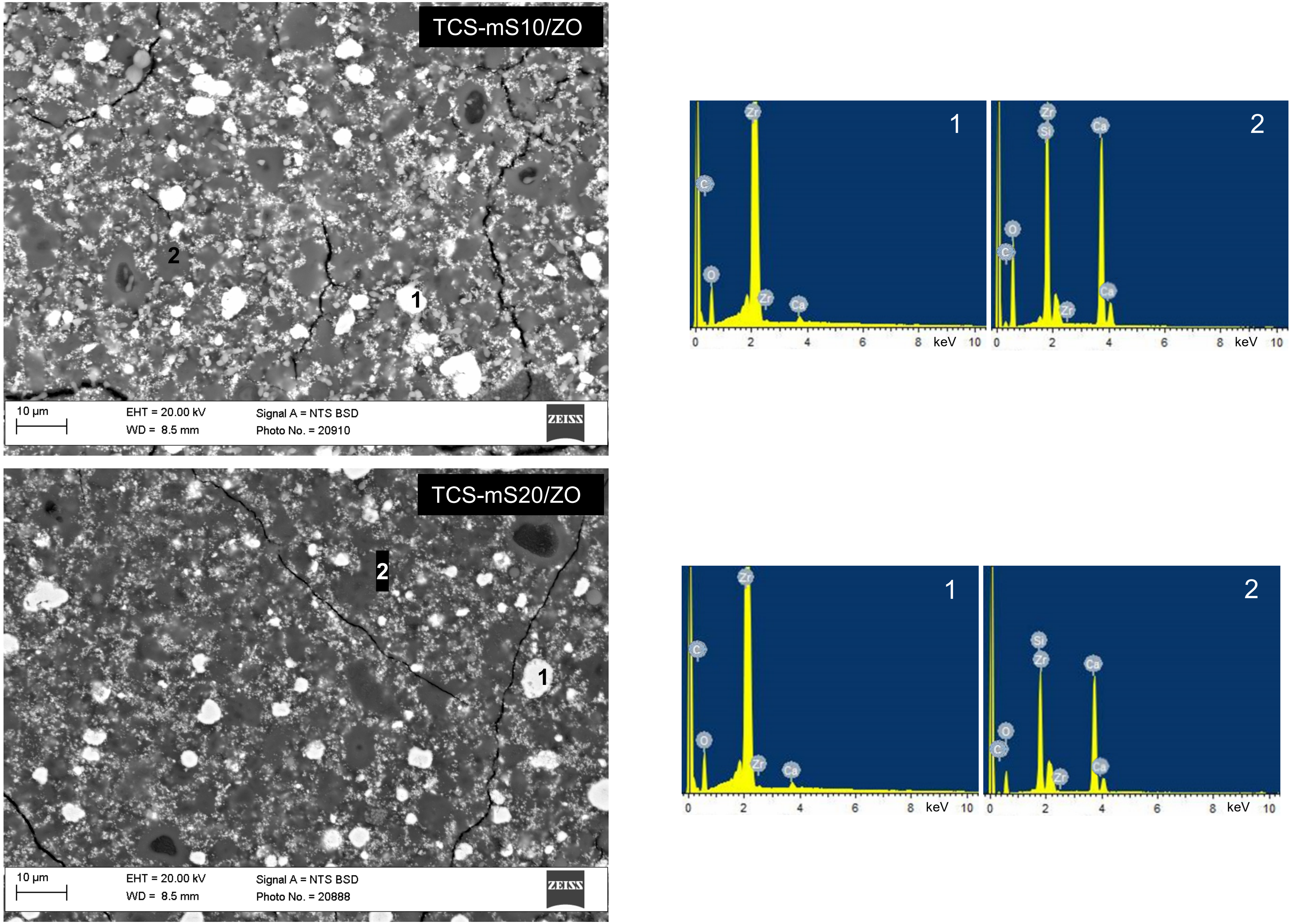

Supplementary Figure S1. Back-scatter scanning electron micrographs of polished sections of commercial and prototype test materials showing microstructural components and energy-dispersive spectroscopic scans of selected areas following a 28-d immersion period in HBSS.
